# Identification and validation of differentially expressed transcripts by RNA-sequencing of formalin-fixed, paraffin-embedded (FFPE) lung tissue from patients with Idiopathic Pulmonary Fibrosis

**DOI:** 10.1186/s12890-016-0356-4

**Published:** 2017-01-12

**Authors:** Milica Vukmirovic, Jose D. Herazo-Maya, John Blackmon, Vesna Skodric-Trifunovic, Dragana Jovanovic, Sonja Pavlovic, Jelena Stojsic, Vesna Zeljkovic, Xiting Yan, Robert Homer, Branko Stefanovic, Naftali Kaminski

**Affiliations:** 1 Department of Biomedical Sciences, College of Medicine, Florida State University, Tallahassee, FL USA; 2 Faculty of Medicine, University of Belgrade, Belgrade, Serbia; 3 Clinic for Pulmonology, Clinical Center of Serbia, Belgrade, Serbia; 4 Institute of Molecular Genetics and Genetic Engineering, University of Belgrade, Belgrade, Serbia; 5 Departement of Thoracopulmonary Pathology, Service of Pathology, Clinical Centre of Serbia, Belgrade, Serbia; 6 Faculty of Medicine, University of Novi Sad, Novi Sad, Serbia; 7 Section of Pulmonary, Critical Care and Sleep Medicine, Yale University School of Medicine, New Haven, CT USA; 8 Department of Pathology, Yale University School of Medicine, New Haven, CT USA; 9 Pathology and Laboratory Medicine Service, VA CT Healthcare System, West Haven, CT USA

**Keywords:** Idiopathic Pulmonary Fibrosis, FFPE, RNA-Seq, Microarray, DEGs, Validation, Pathways, Network, MMP7, NanoString nCounter®

## Abstract

**Background:**

Idiopathic Pulmonary Fibrosis (IPF) is a lethal lung disease of unknown etiology. A major limitation in transcriptomic profiling of lung tissue in IPF has been a dependence on snap-frozen fresh tissues (FF). In this project we sought to determine whether genome scale transcript profiling using RNA Sequencing (RNA-Seq) could be applied to archived Formalin-Fixed Paraffin-Embedded (FFPE) IPF tissues.

**Results:**

We isolated total RNA from 7 IPF and 5 control FFPE lung tissues and performed 50 base pair paired-end sequencing on Illumina 2000 HiSeq. TopHat2 was used to map sequencing reads to the human genome. On average ~62 million reads (53.4% of ~116 million reads) were mapped per sample. 4,131 genes were differentially expressed between IPF and controls (1,920 increased and 2,211 decreased (FDR < 0.05). We compared our results to differentially expressed genes calculated from a previously published dataset generated from FF tissues analyzed on Agilent microarrays (GSE47460). The overlap of differentially expressed genes was very high (760 increased and 1,413 decreased, FDR < 0.05). Only 92 differentially expressed genes changed in opposite directions. Pathway enrichment analysis performed using MetaCore confirmed numerous IPF relevant genes and pathways including extracellular remodeling, TGF-beta, and WNT. Gene network analysis of MMP7, a highly differentially expressed gene in both datasets, revealed the same canonical pathways and gene network candidates in RNA-Seq and microarray data. For validation by NanoString nCounter® we selected 35 genes that had a fold change of 2 in at least one dataset (10 discordant, 10 significantly differentially expressed in one dataset only and 15 concordant genes). High concordance of fold change and FDR was observed for each type of the samples (FF vs FFPE) with both microarrays (r = 0.92) and RNA-Seq (r = 0.90) and the number of discordant genes was reduced to four.

**Conclusions:**

Our results demonstrate that RNA sequencing of RNA obtained from archived FFPE lung tissues is feasible. The results obtained from FFPE tissue are highly comparable to FF tissues. The ability to perform RNA-Seq on archived FFPE IPF tissues should greatly enhance the availability of tissue biopsies for research in IPF.

**Electronic supplementary material:**

The online version of this article (doi:10.1186/s12890-016-0356-4) contains supplementary material, which is available to authorized users.

## Background

Idiopathic Pulmonary Fibrosis (IPF) is a chronic interstitial lung disease of unknown etiology associated with high mortality rates, and increased prevalence with age (14-42.7 cases per 100,000 population) [1]. IPF patients have an overall median survival of approximately 3.5 years from the onset of symptoms. The disease is characterized by progressive scaring of the lung parenchyma that leads to loss of lung function [2]. IPF is thought to result from repeated cycles of alveolar epithelial cell injury leading to fibroblast proliferation, exaggerated accumulation of extracellular matrix in the lung parenchyma and recapitulation of developmental pathways [3, 4].

Currently, IPF diagnosis is based on the exclusion of known causes of lung fibrosis, and the presence of a Usual Interstitial Pneumonia (UIP) pattern on High-Resolution Computed Tomography scan (HRCT) in patients who do not undergo lung biopsy or the combination of a permissive HRCT and the UIP pattern on surgical lung biopsy [5]. The UIP histology pattern is characterized by spatial and temporal heterogeneity, which refers to patchy distribution of dense parenchymal scar along with areas of fibroblast and myofibroblast accumulation and proliferation, known as fibroblastic foci, alternating with areas of less affected or normal lung parenchyma [5, 6]. While lung biopsies were frequently performed to identify the typical UIP pattern as part of the IPF diagnostic workup, the success of HRCT in demonstrating “UIP” radiological patterns has considerably limited the number of lung biopsies currently being performed [7]. That in turn has led to a decline in the availability of tissues for IPF research in general, and for transcriptomic profiling in particular.

Transcriptomic profiling in IPF has largely been performed by microarrays using RNA obtained from whole lung lysates obtained from fresh frozen tissues. These studies have provided significant mechanistic insights regarding IPF pathogenesis, and have largely impacted the field of lung fibrosis [4, 8]. However, these studies are limited because acquisition of fresh frozen tissues is only available in highly specialized academic centers with tissue banking facilities. Thus, the majority of studies contain mostly tissues explanted from patients with IPF at the time of biopsy, and studies containing tissues obtained from diagnostic biopsies are limited. Additionally, it is nearly impossible to assess the lung morphology on frozen tissue, thus the studies utilizing fresh frozen samples depend on histological assessment of adjacent tissue which may or may not contain the exact same pathology. While transcriptomic data generated from different dissections within a single lobe of the lung are highly correlated [9], and that transcriptomic data correlates well with UIP pattern itself [10, 11] the lack of visual confirmation of the histology of the region profiled is still considered a limitation [12].

RNA isolated from Formalin-Fixed Paraffin Embedded FFPE tissues is partially degraded, thus transcriptomic analysis of FFPE tissues was considered challenging [13, 14]. Several recent studies demonstrated that transcriptomic analysis of FFPE tissues using microarrays was possible, was nearly comparable to fresh frozen tissues but still had significant limitations [15–17]. In contrast to microarrays, next generation RNA Sequencing (RNA-Seq) allows for relatively unbiased measurements of expression levels across the entire length of a transcript and its level of expression [18], and therefore may be more suitable for sequencing of partially degraded FFPE RNA. Transcriptomic analysis of FFPE tissues by RNA-Seq demonstrates high concordance to RNA-Seq data produced from matching fresh frozen tissues [19–23]. Because formalin fixation and paraffin embedding is routinely done on all samples from clinically indicated lung biopsies, optimization of a method to perform genome scale transcript profiling of archived FFPE tissues will greatly enhance the access to IPF lungs.

**Fig. 1:**
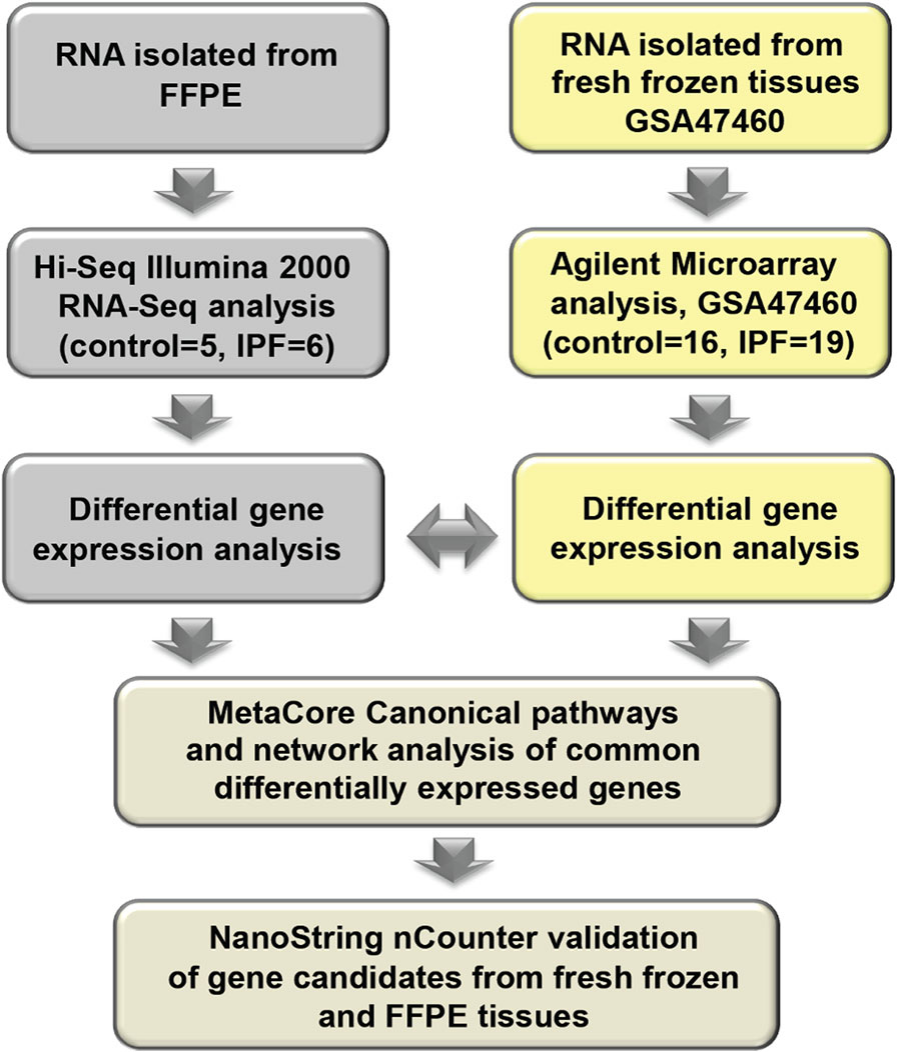
Study design. The summary of study cohorts, sequencing approaches and data analysis. Arrows represent directions of how experiments were performed for each cohort and how comparison between data sets were done. Microarray data is a publically available dataset (GSE47460)

In this study, we sought to determine whether whole transcriptomic analysis of RNA isolated from FFPE biopsies by RNA-Seq was feasible in IPF, and whether the results are comparable to those obtained from gene expression microarrays. To test our hypothesis, we isolated RNA from FFPE lung biopsies of IPF individuals and controls, generated RNA-Seq expression data and compared it to publically available microarray array data previously generated by us from fresh frozen IPF lung tissues (GSE47460, [24]) (Fig. 1). Our study demonstrates high concordance in IPF relevant genes and pathways between RNA-Seq and microarrays of un-paired tissues from patients evaluated in different cohorts, suggesting that RNA-Seq from FFPE tissues could be considered an acceptable technique for transcriptomic profiling in IPF.

## Methods

### FFPE Tissue specimens

Lung FFPE biopsies were obtained from departmental FFPE archives [Clinic for Pulmonology in Belgrade, *n* = 8, and the Lung Tissue Research Consortium (LTRC), *n* = 4] according to Institutional Review Board (IRB) approved study protocols. Informed consents to participate in the study were also obtained according to IRB. The median archival age of FFPE tissues was 6 years. IPF (*n* = 7) lung and control (*n* = 5) FFPE tissues were used for RNA-Seq. Clinical, demographic and histopathological features of the subjects in the study were analyzed by a multidisciplinary group of clinicians and pathologists to confirm IPF diagnosis [5].

### FFPE RNA isolation and quality control

Five 10-μm slices of the whole lung tissue were cut from FFPE block, excess paraffin was trimmed and slices were treated twice with 1ml xylene for 30 min at 62°C, then washed twice with 100% ethanol as previously described [25]. Total RNA was isolated by using MasterPure kit (Epicentre Biotechnologies). The final RNA concentration and purity (A_260_/A_280_) was measured using a NanoDrop ND-1000 spectrophotometer (NanoDrop Technologies). RNA quality and RNA integrity (RIN) was assessed using a 2100 Bioanalyzer (Agilent). For each FFPE tissue block, two consecutive RNA isolations from the whole lung tissue slices (5x10-μm) were performed.

### Fresh frozen tissue specimens

The Lung Genomic Research Consortium (LGRC) contains data for over 200 IPF patients and controls. To have similar size cohorts we picked 35 age and gender matching samples (19 IPF and 16 normal histology samples) and reanalyzed the data. The experiments were approved by the Institutional Review Board. These samples are publically available at GSE47460 and at the LGRC website - http://www.lung-genomics.org) and have been previously described at [24, 26].

### RNA-Seq library preparation and paired-end RNA-Seq

Approximately 1.5μg of total RNA was isolated from each FFPE block (tissues size ~10mm x 7mm). FFPE RNA showed fragmentation in a range of ~100-150bp. To increase the depth of RNA-Seq sequencing and mapping rate of sequencing reads [19, 27], ribosomal RNA was removed by using the RiboZero rRNA removal kit (Epicentre) prior to cDNA library preparation. Double stranded cDNA library was prepared by using NEBNext® Ultra™ Directional RNA Library Prep kit for Illumina (New England Biolabs) following manufacturer’s protocol from 1ug of total RNA. Kappa cDNA library quality control was performed prior to pooling libraries for flow cell amplification. All cDNA libraries were sequenced using Illumina HiSeq 2000 to produce 50 million reads, 50bp paired-end reads with multiplexing (4 samples/lane) (cDNA library preparation and RNA-Seq run was performed by Genomic Service Lab at Hudson Alpha).

### RNA-Seq reads processing and alignment

TopHat2 [28] was used to map the sequencing reads to the human genome (UCSC hg19) by allowing multiple hits. Mapping rate was calculated as the percentage of read that were properly mapped. Samples with low mapping rates (<20%) were discarded from further analysis. Cufflinks 2.2.0 [29] was used to calculate FPKMs values as the estimated gene expression levels. FPKMs between IPF and controls were compared using Cuffdiff and genes with a false discovery rate (FDR) adjusted *p* < 0.05 were identified as differentially expressed genes (DEGs) [30]. Cuffdiff test assigns a status of genes as: OK - test successful, NOTEST - not enough alignments for testing, LOWDATA - too complex or shallowly sequenced, HIDATA - too many fragments in locus, or FAIL - an ill-conditioned covariance matrix or other numerical exception prevents testing [31]. In this study we report genes with OK status as genes with sufficient coverage (Additional file 1: Table S1). Multidimensional scaling (MDS) analysis was performed for data visualization [32].

### Microarray data

We used previously generated microarray data (Agilent). Briefly, the normalization of the gProcessed signal was performed using cyclic-LOESS and bioconductor package. Complete datasets and protocols were previously published [24, 26], and deposited in data repository GEO (accession no. GSE47460), and are also available in the Lung Genomics Research Consortium’s (LGRC) website (http://www.lung-genomics.org/). Fold change and FDR values were calculated using the Significance Analysis of Microarrays (SAM) tools. Microarray experiments were compliant with MIAME guidelines.

### Comparison of IPF signatures obtained from FFPE RNA-Seq and from microarray analysis of fresh frozen tissue

Considering that nearly all of the transcriptomic data in IPF was generated on fresh frozen tissues we compared IPF signatures obtained with FFPE RNA-Seq (Illumina) with those obtained from Microarray (Agilent) gene expression data. Unique gene probes from microarrays (*n* = 16,741) were matched with genes having sufficient coverage by RNA-Seq (*n* = 15,149) by the gene symbol which provided a matched set of 13,304 genes. For analysis of differentially expressed genes, Significance Analysis of Microarrays (SAM) was used in the case of microarrays, and Cuffdiff was used in the case of RNA-Seq. Significance was defined as FDR adjusted *p* < 0.05. A discordant gene was defined as a significant gene that was increased in microarray but decreased in RNA-Seq and vice versa. The fold change (FC) of each gene was calculated by dividing the average mean value of IPF group by the average mean value of control group for both RNA-Seq and microarray. Log_2_FC is the log base 2 transformed FC. Log_2_FC of microarray were plotted against Log_2_FC of RNA-Seq values for matching genes between the two platforms.

### Pathway enrichment analyses

Pathway enrichment analysis of the common Differentially Expressed Genes (DEGs) between the two techniques, RNA-Seq and Microarrays, was performed using MetaCore (Thomson Reuters). In this way, we identified the top 13 statistically significant enriched pathways with FDR adjusted p value <0.1 for pathways of common increased genes, and the top 50 statistically significant enriched pathways with FDR adjusted *p* < 0.1 for pathways of common decreased genes. Gene candidates with fold change > 1 from top pathways were presented in the heatmap to show the distribution of IPF relevant genes among the pathways. To analyze the interaction between members of the gene network in RNA-Seq and microarray data independently, networks were built around MMP7, one of the most-widely studied IPF-relevant genes [33]. When building the network, we used all RNA-Seq (4,131) and all microarray (5,859) differentially expressed genes. With auto-expand and canonical options, the network was built and drawn by plotting genes from datasets to pre-built MetaCore network for MMP7. Members of the gene networks with their expression values and interactions were compared.

### NanoString nCounter® gene expression quantification and validation

100ng of RNA isolated from fresh frozen lung tissues and 250ng of RNA isolated from FFPE lung tissues, as suggested by the NanoString protocol, were used in experiment. 7 CTRL and 8 IPF FF samples, and 5 CTRL and 7 IPF FFPE samples were used for validation. Probe set for each gene were designed and synthesized by NanoString nCounter®. For validation, we focused on genes that had at least two-fold change in at least one dataset. Thus we selected 10 discordant genes, 10 genes that were significantly differentially expressed only in one dataset and 15 concordant genes (Additional file 2: Table S5). The discordant genes were: IFNG, BCL11B, PDPR, ADAM33, VPS13B, TNRC18, PPBP, PHYHIP, KRT14, ITLN1. The differentially expressed genes in only one dataset were: MMP1, COL1A1, SERPIND1, ADAM23, COL1A2, MMP9, WISP1, WNT2, FOS, FGG. The concordant genes were: SERPINE1, CXCL2, MMP19, CAV1, CTNNA1, WNT10A, MMP7, SPP1, CXCL13, PLA2G2A, POSTN, LAMA3, COL17A1, SLC6A4, ANKRD1. We used RNA isolated from 7 IPF and 5 CTRL FFPE tissues and RNA isolated from 8 IPF and 7 CTRL fresh frozen tissues from the LGRC cohort. We have followed a standard manufacturer protocol for sample preparation, hybridization and detection. Data were analyzed using nSolver 3.0 digital analyzer software.

## Results

### Quality of RNA isolated from FFPE tissues

Consistent with previous reports [15, 25] the integrity of RNA isolated from FFPE tissues was decreased probably due to formalin fixation and archival time. All RNA isolations had OD_260/280_ > 1.9 confirming the high purity of RNA (data not shown). The RIN numbers were in the range of 2.1–2.6 and the most abundant RNA fragments were in the range of 100–150 ribonucleotides for all samples (Additional file 3: Figure S1) suggesting a similar degradation and quality regardless whether FFPE tissues were obtained from controls or IPF patients. Repeat isolations of RNA per FFPE block had very similar fragmentation and the same RIN number (Additional file 3: Figure S1). Archival time had no effect on RNA quality. We proceeded with one of the RNA isolation per FFPE block for cDNA library preparations and RNA-Seq analysis.

### Mapping, transcript quantification and analysis of differentially expressed genes of FFPE RNA-Seq data

**Fig. 2:**
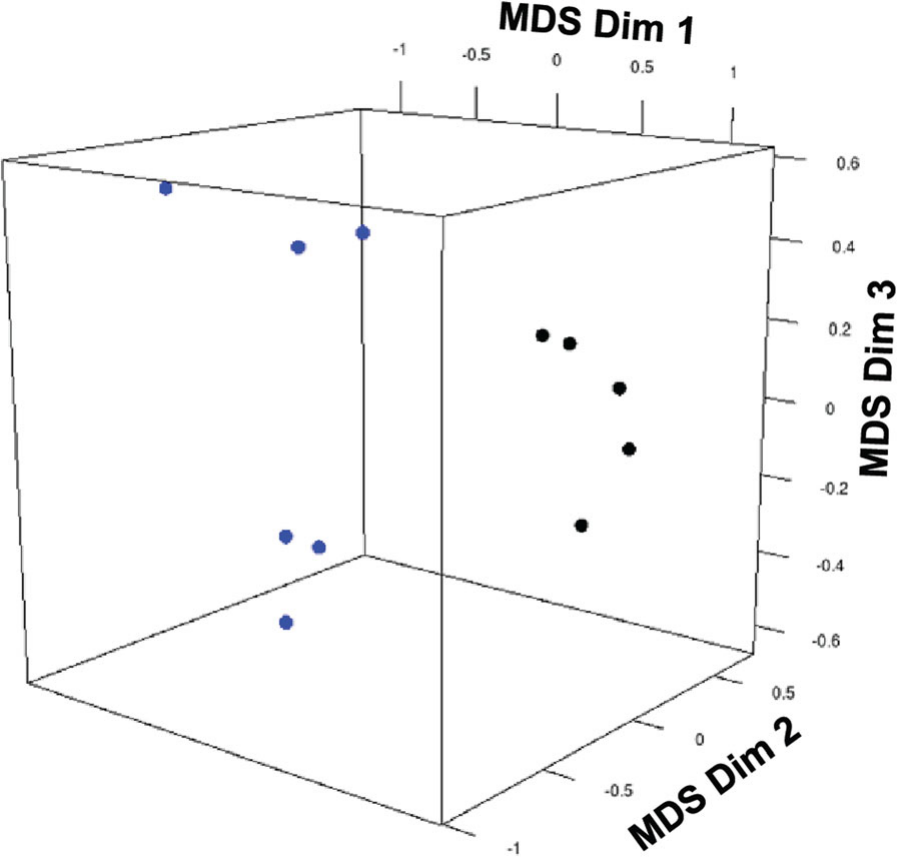
MDS analysis based on gene expression demonstrates a clear separation between the IPF and control FFPE samples. The top three MDS dimensions based on the top 5,000 genes differentially expressed between IPF and control were plotted using edge R package for data visualization. Each dot is one sample. Blue represents IPF and black represents control, respectively

Mapping sequencing reads to the human genome produced an average of ~116 million reads at 50bp per sample with ~ 62 million mapped reads (mapping rate 48%). Only one RNA sample, corresponding to FFPE 7 (Additional file 3: Figure S1) had a lower mapping rate, 20.94% corresponding to 15.9 million reads (Table 1). This sample was excluded from downstream analysis. While we did not observe an effect of archival time on RIN number, samples with archival time longer than 7 years had lower mapping rates (40–50 million reads), a finding consistent with previous reports [21]. RNA–Seq identified 15,149 genes with sufficient coverage out of the 23,615 annotated genes in hg19, after filtering out genes that did not have enough alignments for testing, were too complex, had low number of sequencing reads or had too many fragments in locus. Out of those 15,149 genes, Cuffdiff identified 4,131 differentially expressed genes (FDR < 0.05), including 1,920 increased genes and 2,211 decreased genes (Additional file 1: Table S1). Multidimensional scaling (MDS) analysis demonstrated a clear separation between control and IPF FFPE samples based on expression profiles (Fig. 2).

**Table 1:** Summary of RNA-Seq (FFPE) gene mapping

FFPE	RNA Seq	Orig Reads	QC failed reads	Unmapped Reads	Mapped Reads	Hits	Proper hits	Mapping Rate	Hits Rate	Proper hits Rate
1	SL32670	109112224	1585386	55426188	52100650	61090707	49057850	0.4775	0.5599	0.4496
2	SL32671	112024858	1901325	65686848	44436685	52541559	40745436	0.3967	0.469	0.3637
3	SL32674	137705852	1225400	58878848	77601604	93224772	74057010	0.5635	0.677	0.5378
4	SL32677	114532074	3536592	65895279	45100203	52390752	36239592	0.3938	0.4574	0.3164
5	SL32679	94880502	1404145	41223487	52252870	60762016	46203712	0.5507	0.6404	0.487
6	SL32680	98383774	3001192	45024767	50357815	58405722	44600578	0.5119	0.5937	0.4533
7	SL32681	76149832	2406659	57793748	15949425	17505889	8309682	0.2094	0.2299	0.1091
1C	SL32683	191953246	1025564	44983418	145944264	1.72E + 08	135808266	0.7603	0.8955	0.7075
2C	SL71047	126656896	4828921	72935707	48892268	59052405	40219390	0.386	0.4662	0.3175
3C	SL71048	92915968	5523822	45167979	42224167	51054887	38389264	0.4544	0.5495	0.4132
4C	SL71049	130059384	8904855	55527773	65626756	82086047	60494026	0.5046	0.6311	0.4651
5C	SL71050	121812940	7765072	43474914	70572954	83920938	60927332	0.5794	0.6889	0.5002

### Comparison of differentially expressed genes of FFPE RNA-Seq to Microarray analysis of fresh frozen tissues

**Fig. 3:**
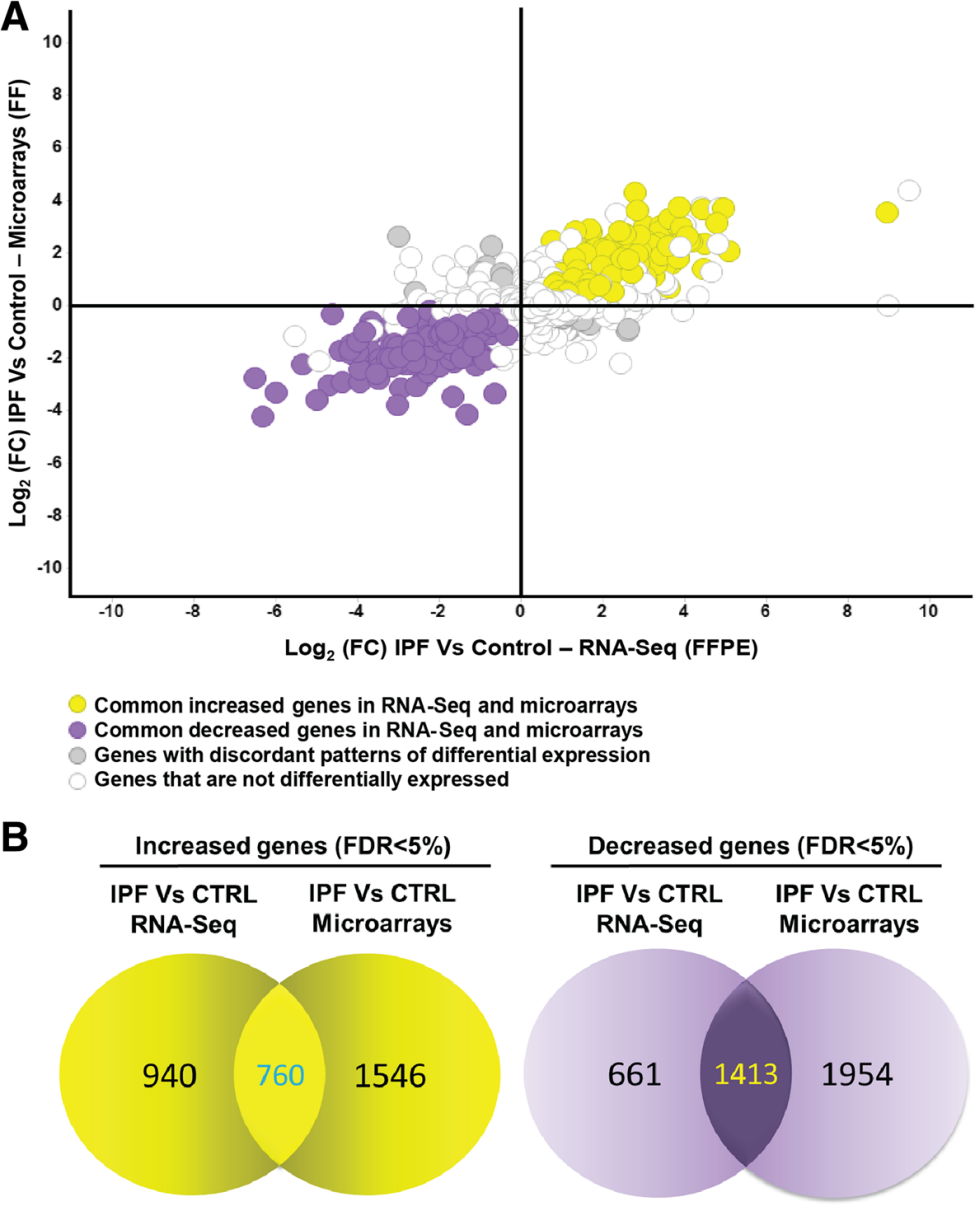
Direct comparison of gene expression between RNA-Seq (FFPE) and microarray data (FF). **a** Microarray Log_2_(FC) IPF vs control was plotted on x axis and RNA-Seq Log_2_(FC) IPF vs control was plotted on y axis. Yellow dots indicate common increased genes, purple dots indicate common decreased genes, grey dots indicate genes with discordant patterns of differential expression and white dots indicate genes that are not significantly differentially expressed genes in both datasets or not significant in microarray and significant in RNA-Seq and vice versa. **b** Venn diagram colored in yellow indicates gene overlap between increased genes in RNA-Seq and microarray. 760 represents commonly increased genes, 940 is a number of genes that is increased in RNA-Seq data and do not overlap with microarrays while 1,546 is a number of increased genes in microarrays that do not overlap with RNA-Seq (FDR adjusted *p* < 0.05). Venn diagram colored in purple represents overlap between of decreased genes in both sets (FDR adjusted *p* < 0.05) and it follows the same logical relations as a Venn diagram in yellow

The IPF expression profile has been well defined by microarray analysis of RNA isolated from fresh frozen IPF lung tissues [34–36]. To validate gene expression profiles obtained from FFPE IPF tissues, we compared it to gene expression data from microarrays based on RNA isolated from fresh frozen lungs (GSE47460, [24]). Figure 3a, depicts the comparison of microarray and RNA-Seq datasets. Microarray analysis demonstrated 2,306 increased and 3,367 decreased genes (FDR <0.05) between IPF and controls while FFPE RNA-Seq identified 1,920 increased genes and 2,211 decreased genes. 760 increased and 1,413 decreased genes overlapped between microarrays and FFPE RNA-Seq (Fig. 3a, yellow and purple dots; Fig. 3b, Additional file 4: Table S4). Only 92 genes that were significantly differentially expressed were discordant between platforms (Fig. 3a, grey dots). 11,039 genes (Fig. 3a, white dots) were not differentially expressed (FDR >0.05) in both datasets. 940 and 1,546 increased genes and 661 and 1,954 decreased genes that did not overlap between FFPE RNA-Seq and microarrays (Fig. 3b, white dots on Fig. 3a). To determine whether the overlap between the differentially expressed genes in FFPE RNA-Seq and FF microarrays in both datasets was not due to random association, we performed a hypergeometric test which revealed that the overlap was highly significant (*p* < 10^-182^) for both increased and decreased genes. The hypergeometric test for discordant genes between FFPE RNA-Seq and FF microarrays revealed a probability of p~1, suggesting that discordant genes are identified due to random association.

### FFPE RNA-Seq results contain IPF relevant biological information

**Fig. 4:**
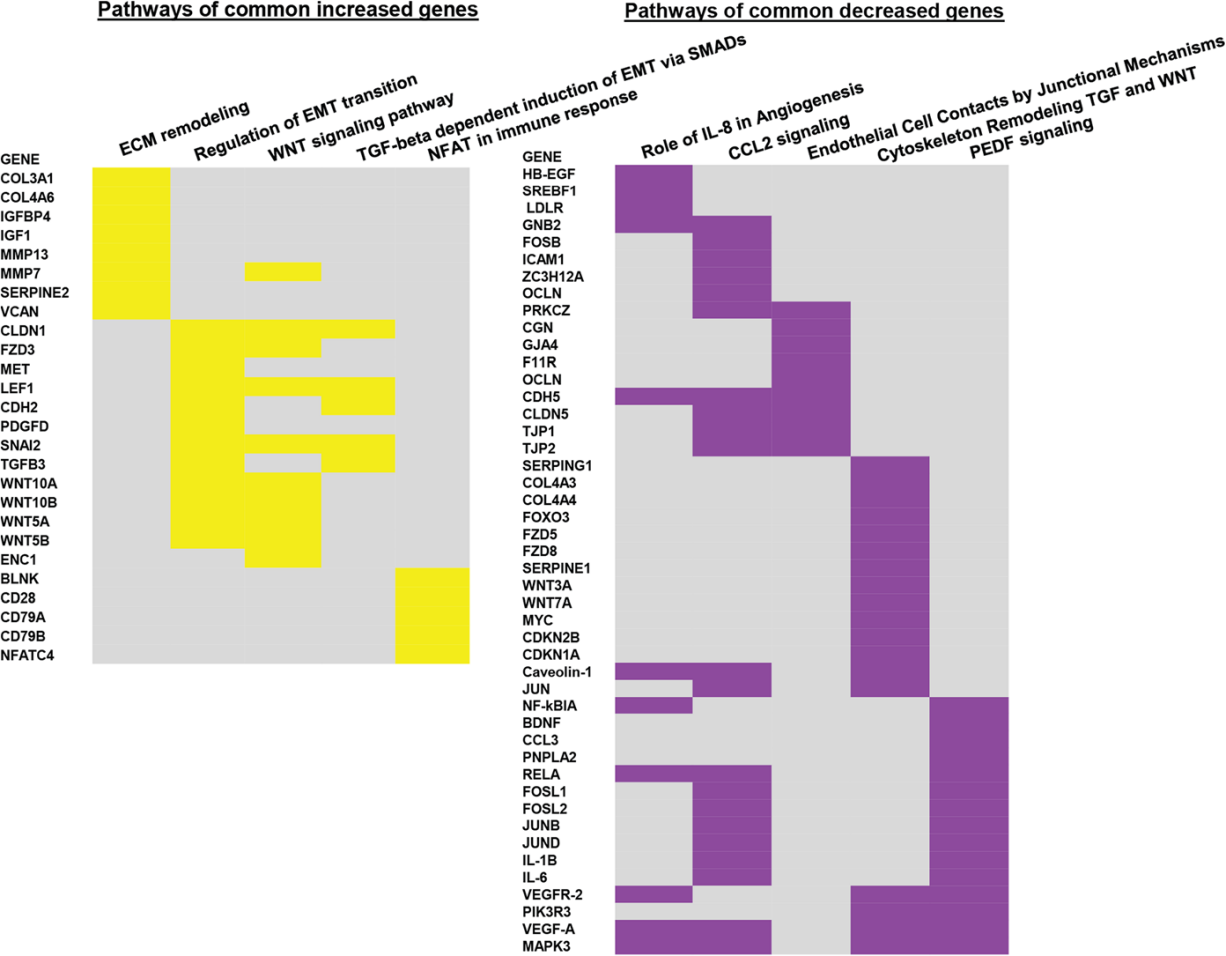
Heat map of top scored signaling pathways enriched in commonly increased and decreased genes from RNA-Seq (FFPE) and microarrays (FF). Every raw represents a gene and every column represents a signaling pathway. Top significant signaling pathways for commonly increased genes are presented on the heat map in yellow and for decreased genes are presented on the heat map in purple. Pathway enrichment analysis was done in MetaCore and full list of pathways could be found in Additional file 5: Table S2 and Additional file 6: Table S3. Only genes that have fold change above one were presented in the heat map

**Fig. 5:**
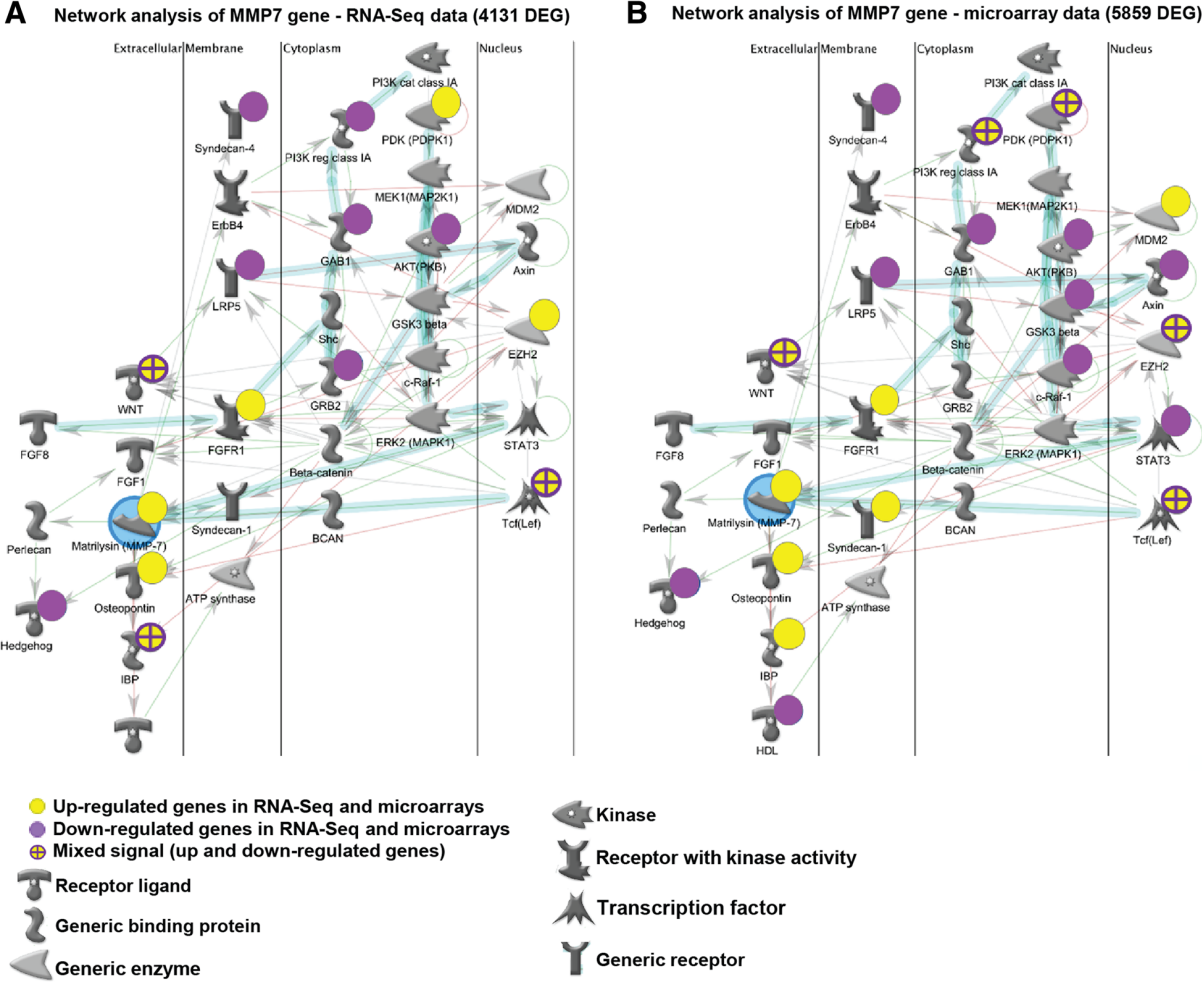
MMP7 network analysis from RNA-Seq (FFPE) and microarrays (FF) data independently. All differentially expressed genes from RNA-Seq (4,131) and from microarrays (5,859) were submitted to MetaCore to build and draw the network around MMP7 gene, a common network gene for both datasets. Increased genes were marked in yellow and decreased genes were marked in purple for both datasets (RNA-Seq (**a**), and microarrays (**b**)). Gene homologues that have mixed expression values were marked with yellow/purple*. The rest of the genes that are present in network but are not detected in our datasets, belong to a pre-build network for MMP7 in MetaCore database. Canonical pathways identified in network are marked in light blue. Red arrows represent inhibitory effect between two genes in the network and green arrows represent activation effect. Function of each network gene is defined by different shape and explained in the figure legend. *Please note yellow/purple colors were manually added, instead of red/blue originally proposed by MetaCore, to keep consistent gene expression visualization through the manuscript

Previous studies on IPF gene expression profiles identified gene candidates and significant pathways directly involved in IPF development [34, 36–39]. To investigate whether IPF relevant genes and pathways could be detected in our RNA-Seq data, we analyzed canonical pathways and IPF relevant gene networks of RNA-Seq and microarray overlapping genes by MetaCore (Fig. 4). Among the top 50 pathways significantly associated with our dataset (Additional file 5: Table S2 and Additional file 6: Table S3) we found many pathways known to play a significant role IPF [8, 37, 39] including developmental, cytoskeleton, extracellular remodeling and cell adhesion pathways. We performed a cluster analysis of genes that had a fold change above one and were present in at least one pathway. Figure 4 (left panel) represents a summary of increased genes in the following top five increased pathways: Extracellular matrix remodeling, regulation of EMT transition, WNT, TGF-β and NFAT pathways. Figure 4 (right panel) provides a summary of decreased genes in top five decreased pathways: IL8, endothelial cell contacts, CCL2, cytoskeletal remodeling TGF/WNT, and PEDF signaling. We also identified several genes such as: COL3A1, COL4A6, MMP7 and MMP13, TGF-beta, WNT family, Serpine 1, LEF1, CLDN1 and CAV1 which had been shown to be relevant to IPF pathogenesis [33, 36, 40–44] suggesting that IPF relevant genes could be detected in FFPE tissues. To further support the notion that expression profiles from FFPE tissues are a valid source of information for transcriptomic profiling in IPF, we performed a network analysis for MMP7 gene, a well-known IPF relevant gene [24, 33, 45]. We hypothesized that building a network around MMP7 gene, should allow us to see if two datasets predict the same networking candidates and directions of interaction between candidates. For this purpose we performed an independent MetaCore network analyses using all RNA-Seq (4,131) and microarray (5,859) differentially expressed genes. Figure 5 shows that out of a total of 33 gene candidates proposed for RNA-Seq MMP7 network (Fig. 5a) and microarray MMP7 network (Fig. 5b), 15 genes were identified in RNA-Seq data and 21 genes were identified in microarray data. 14 genes overlapped between networks and 11 genes were not identified in any dataset. 7 genes in the MMP7 network (GSK3, c-Raf1, MDM2, Axin, Stat3, Syndecan 1, HDL) were differentially expressed in microarray data (with less than two fold change), but not in RNA-Seq data. Only one gene, GRB2, was differently expressed in RNA-Seq data (with less than two fold change) but not in microarray data. The 14 genes that overlap between two MMP7 networks represent 67% (14 out of 21) of all gene candidates for MMP7 network identified in our data and have preserved directions of interactions with surrounding genes. The hypergeometric test on 14 genes that overlap between microarray and RNA-Seq in network analysis revealed *p* = 2.6x10^-30^ for RNA-Seq and *p* = 1.4x10^-28^ for microarray confirming that the gene overlap is highly significant. This demonstrates that FFPE data provides significant gene network information that is comparable to the gene network obtained from un-paired fresh tissues.

### NanoString nCounter® gene expression validation in fresh frozen and FFPE tissues

**Fig. 6:**
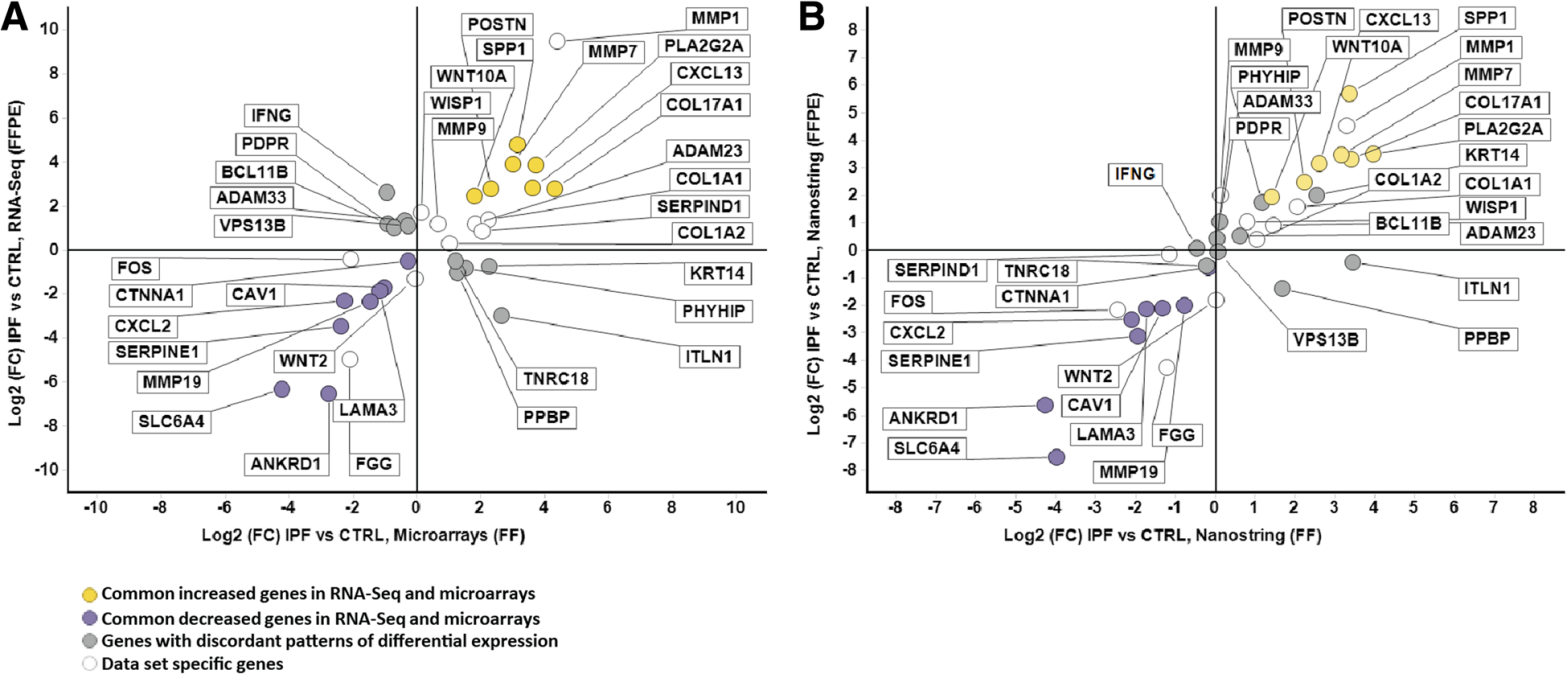
Validation of gene expression in fresh frozen and FFPE tissues using NanoString nCounter®. **a** Microarrays Log_2_(FC) IPF vs control (FF) was plotted on x axis and RNA-Seq Log_2_(FC) IPF vs control (FFPE) was plotted on y axis, **b** NanoString Log_2_(FC) IPF vs control (FF) was plotted on x axis and NanoString Log_2_(FC) IPF vs control (FFPE) was plotted on y axis. 15 concordant genes, 10 discordant genes, and 10 data set specific genes were analyzed. Gene names and categories are labeled

We performed validation of gene expression levels by NanoString nCounter® technology. This technology performs better than RT-PCR in archived FFPE tissues [46]. Overall NanoString nCounter® results correlated well with both microarrays (r = 0.92) and RNA-Seq (r = 0.90). Detailed results for all genes are provided in Additional file 2: Table S5. The fold change directionality of all 15 concordant genes was confirmed by NanoString (Fig. 6a, b). Out of 10 discordant genes, only 4 genes (IFNG, ITLN1, PPBP, VPS13B) remind discordant after NanoString validation (Fig. 6b and Additional file 7: Figure S2, lower panel). Out of 10 tested genes that were significantly expressed in at least one dataset only 2 genes from microarray (SERPIND1, WNT2) and 1 gene from RNA-Seq (SERPIND1) were not confirmed (Fig. 6b, and Additional file 7: Figure S2, lower panels). Overall, we validated the significant changes for most genes for each type of samples (FF vs FFPE). This suggests that the source of discordance may have been tissue heterogeneity and samples being un-paired.

## Discussion

Our study demonstrates that transcriptomic analysis of RNA isolated from FFPE IPF lung biopsies by RNA-Seq is feasible and the results comparable to those obtained from gene expression microarrays. RNA-Seq resulted in an average of ~116 million 50 bp reads per sample with average of ~ 62 million mapped reads (Table 1). Our depth of sequencing, 62 million mapped reads, allowed for the detection of total of 15,149 genes with sufficient coverage, out of which 4,131 were differentially expressed between IPF and control (FDR adjusted *p* < 0.05). To validate the RNA-Seq FFPE results, we compared them to gene expression microarrays obtained from FF tissues and identified overlapping differentially expressed genes. The overlap was statistically significant. The common genes were enriched for signaling pathways relevant to IPF such as: ECM remodeling process, WNT, TGF-β, NFAT, IL-8 in angiogenesis, CCL2 signaling and PEDF signaling [8, 36, 38, 39] and network analyses of both datasets revealed similar networks suggesting that FFPE RNA-Seq generated information that was relevant to IPF and comparable if not perfectly identical to FF tissue. Validation by NanoString nCounter® of concordant, discordant and dataset specific genes largely confirmed the results. Taken together, these findings demonstrate the feasibility and validity of RNA-Seq FFPE data and its relevance to IPF.

Although we provide strong evidence about the validity of our transcriptome RNA-Seq analysis, there are several discrepancies present when comparing RNA-Seq and microarray data. In addition to detecting the commonly expressed genes, we also detected differentially expressed genes that are discordant or do not overlap between RNA-Seq and microarray (Fig. 3b, gray and white dots on Fig. 3a, Additional file 4: Table S4). Computationally, we used MMP7 as a model gene to build the gene network and assess the potential bias in detecting gene interacting candidates from two datasets due to the presence of discordant or non-overlapping genes. For network analysis, we took into consideration all differentially expressed genes in RNA-Seq (4,131) and in microarray (5,859) independently. Out of 15 differentially expressed genes from RNA-Seq and 21 differentially expressed genes from microarray that were found in the MMP7 network, 14 overlapped between datasets. 7 genes were only differentially expressed in microarray and one in RNA-Seq (Fig. 5). These 8 genes do not overlap between datasets (Fig. 3b, white dots on Fig. 3a) suggesting differences between datasets. To validate the results experimentally, and determine whether the results of comparison of different methodologies (microarrays vs RNA-Seq), or sample type (un-paired, FF vs FFPE) or other reasons we validated expression 35 genes: 10 discordant genes, 15 concordant genes, and 10 genes significant in only one dataset, using the NanoString nCounter®, a high precision system that measures gene expression based on digital color-coded barcode technology that provides significant accuracy and sensitivity and has been used successfully in partially degraded RNA samples. For most of the genes, directionality and significance of gene expression changes was confirmed. The number of discordant genes has been decreased with Nanostring nCounter® suggesting that differences in methodologies accounted for at least some of the differences.

The most significant limitations of our study are the small number of samples and the fact that we did not have paired FFPE and FF tissue from the same anatomical location in the same patient. Despite these limitations we found a significant overlap in differentially expressed genes in both tissue types, a significant overlap in functional annotations of the functional annotations of these genes, and good correlation between our Nanostring nCounter® validation with either microarray analysis of FF tissue or RNA-Seq analysis of FFPE tissue. Our results are in agreement with recent observations that RNA-Seq analysis of FFPE tissues can generate valid and comparable gene expression to FF tissues [16, 20, 27]. In cancer tissues high correlation was observed between RNA-Seq of paired FF and FFPE tissues [19–22, 27, 47]. Important to note, that in all of those studies authors mention that while the information derived from FF or FFPE tissue is comparable, comparisons should be limited within one type of tissue processing. Direct comparison of FFPE diseased tissues to control FF tissues for example, would be highly confounded and probably generate spurious results.

The RNA we isolated from FFPE tissues was partially degraded as previously observed [13–17, 19, 25]. However, while we used FFPE biopsies that had a wide range of archival times, and handling performed at different hospitals, we did not find systematic differences between the tissues. Of 12 FFPE biopsies, we experienced low number of original reads and low mapping rate of only one FFPE sample was indistinguishable from other samples. Archival age, RNA and cDNA quality were similar to the other samples. This could be the result of conditions that cannot be directly observed such as minor changes in fixation technique, or storage that could induce changes in the RNA structures [21]. Thus, in our relatively small study the technical success of RNA-Seq from FFPE tissue was 92%. It is plausible, that in prospectively designed studies and standardized fixation protocols the success rate would be even higher [48].

To the best of our knowledge, our study is the first to demonstrate the feasibility of RNA-Seq of FFPE IPF lung samples. We hope and believe that the availability of our protocols, as well as our results, will facilitate the use of FFPE tissue for genome scale transcript profiling of IPF. This will overcome the limitation on availability of FF tissues and increase the capacity for transcriptomic profiling of IPF [47, 49, 50].

## Conclusion

Our study serves as a proof of principle, that RNA-Seq performed on RNA isolated from archival FFPE IPF lung tissues is feasible, and reveals a gene expression profile relevant for IPF. This study further shows that there is a high concordance between RNA-Seq (FFPE) and microarray (FF) expression profiles for biopsies performed on different patients, and at different hospitals encouraging the further usage of FFPE biopsies. Taking into consideration the great potential for transcriptomic research, FFPE tissues should be considered to overcome limitations in the availability of FF human lung tissues.

## Additional files

Additional file 1: Table S1. FPKM p-value matrices. (XLS 15268 kb)
Additional file 2: Table S5. NanoString validation. (XLS 71 kb)
Additional file 3: Figure S1. Quality of RNA isolated from control and IPF FPPE lung tissues. To analyze RNA isolated from five 10-μm slices of the whole FFPE lung tissue, RNA samples were run on the 2100 Bioanalyzer. RNA ladder was run to determine the size of RNA fragments. Two isolations of RNA per FFPE block, for control and IPF lung tissues, were performed. RIN numbers are presented at the panels. (PNG 632 kb)
Additional file 4: Table S4. IPF vs CTRL, RNA-Seq vs Microarrays, uploaded to Spotfire. (XLS 23589 kb)
Additional file 5: Table S2. Enrichment pathway analysis MetaCore_common decreased genes_microarrays vs RNA-Seq. (XLS 68 kb)
Additional file 6: Table S3. Enrichment pathway analysis MetaCore_common increased genes_microarrays vs RNA-Seq. (XLS 55 kb)
Additional file 7: Figure S2. Detailed results of NanoString nCounter validation. Upper 3 panels. Microarrays Log_2_(FC) IPF vs control (FF) was plotted on x axis and RNA-Seq Log_2_(FC) IPF vs control (FFPE) was plotted on y axis, than Nanostring Log_2_(FC) IPF vs control (FF) was plotted on y axis and Microarrays Log_2_(FC) IPF vs control (FF) was plotted on x axis, than Nanostring Log_2_(FC) IPF vs control (FFPE) was plotted on y axis and RNA-Seq Log_2_(FC) IPF vs control (FFPE) was plotted on x axis. 15 concordant genes between microarray and RNA-Seq were validated with NanoString. Gene names are labeled. FDR and p values are also labeled for each gene and technology. Similar to above, middle 3 panels present 10 discordant genes, and lower 3 panels present 10 genes in specific data set. (PNG 372 kb)

## ᅟ

DEG: Differentially expressed gene
FC: Fold change
FFPE: Formalin fixed paraffin embedded
FPKM: Fragments per kilobase of exon per million
HRCT: High-resolution computed tomography
IPF: Idiopathic pulmonary fibrosis
IRB: Institutional Review Board
LGRC: The lung genomic research consortium
MDS: Multidimensional scaling
MIAME: Minimum information about a microarray experiment
NGS: Next generation sequencing
OD: Optical density
RIN: RNA Integrity number
RNA-Seq: RNA sequencing
RNA: Ribonucleic acid
rRNA: Ribosomal RNA
SAM: Significance analysis of microarrays
UIP: Usual interstitial pneumonia
