# Supplementary figures and images for: Identification and validation of differentially expressed transcripts by RNA-sequencing of formalin-fixed, paraffin-embedded (FFPE) lung tissue from patients with Idiopathic Pulmonary Fibrosis
Source: BMC Pulm Med. 2017 Jan 12;17:15. doi: 10.1186/s12890-016-0356-4 (PMC5228096; doi:10.1186/s12890-016-0356-4)

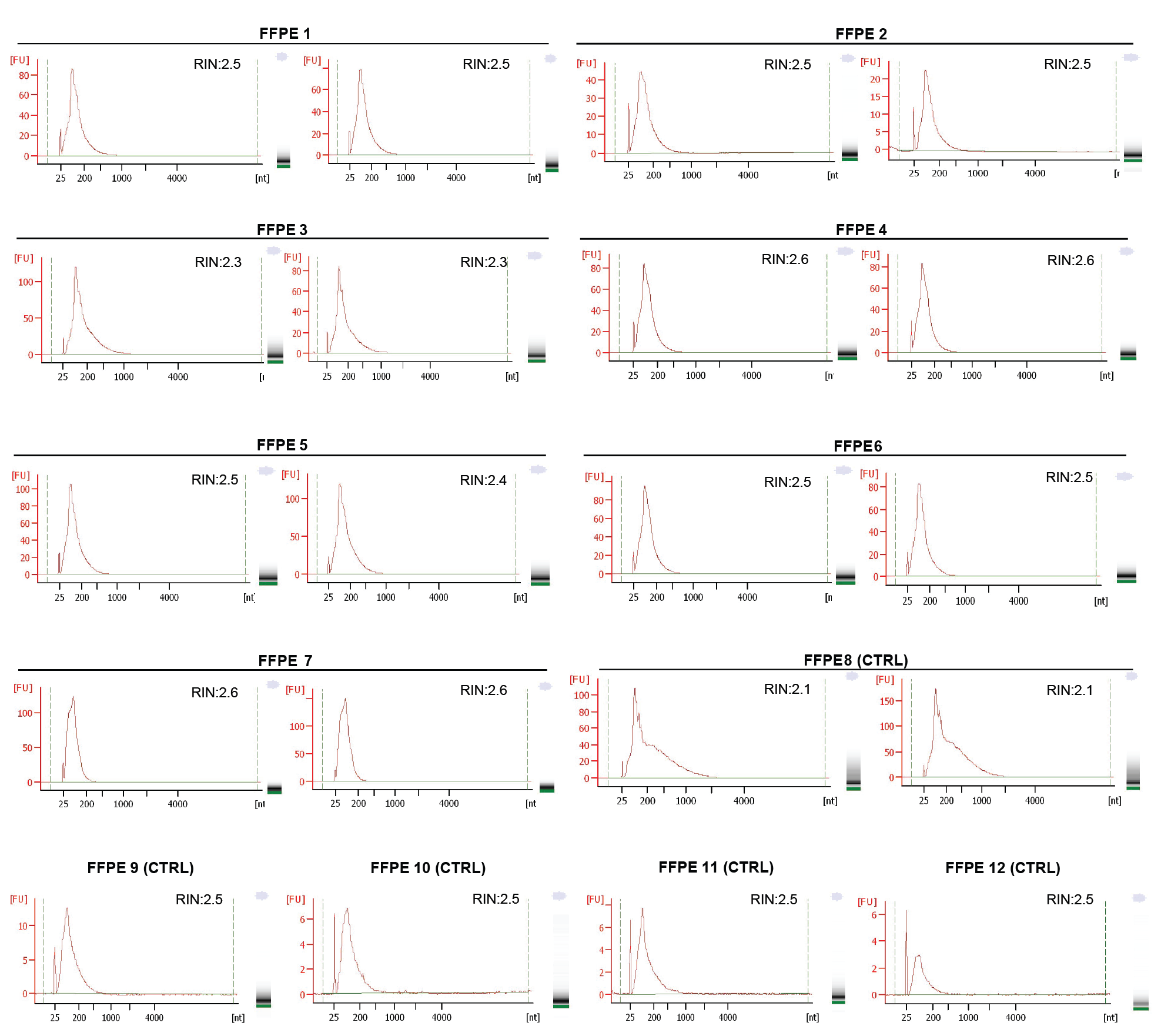

Supplement: Additional file 3: Figure S1. — Quality of RNA isolated from control and IPF FPPE lung tissues. To analyze RNA isolated from five 10-μm slices of the whole FFPE lung tissue, RNA samples were run on the 2100 Bioanalyzer. RNA ladder was run to determine the size of RNA fragments. Two isolations of RNA per FFPE block, for control and IPF lung tissues, were performed. RIN numbers are presented at the panels. (PNG 632 kb) [file 12890_2016_356_MOESM3_ESM.png]

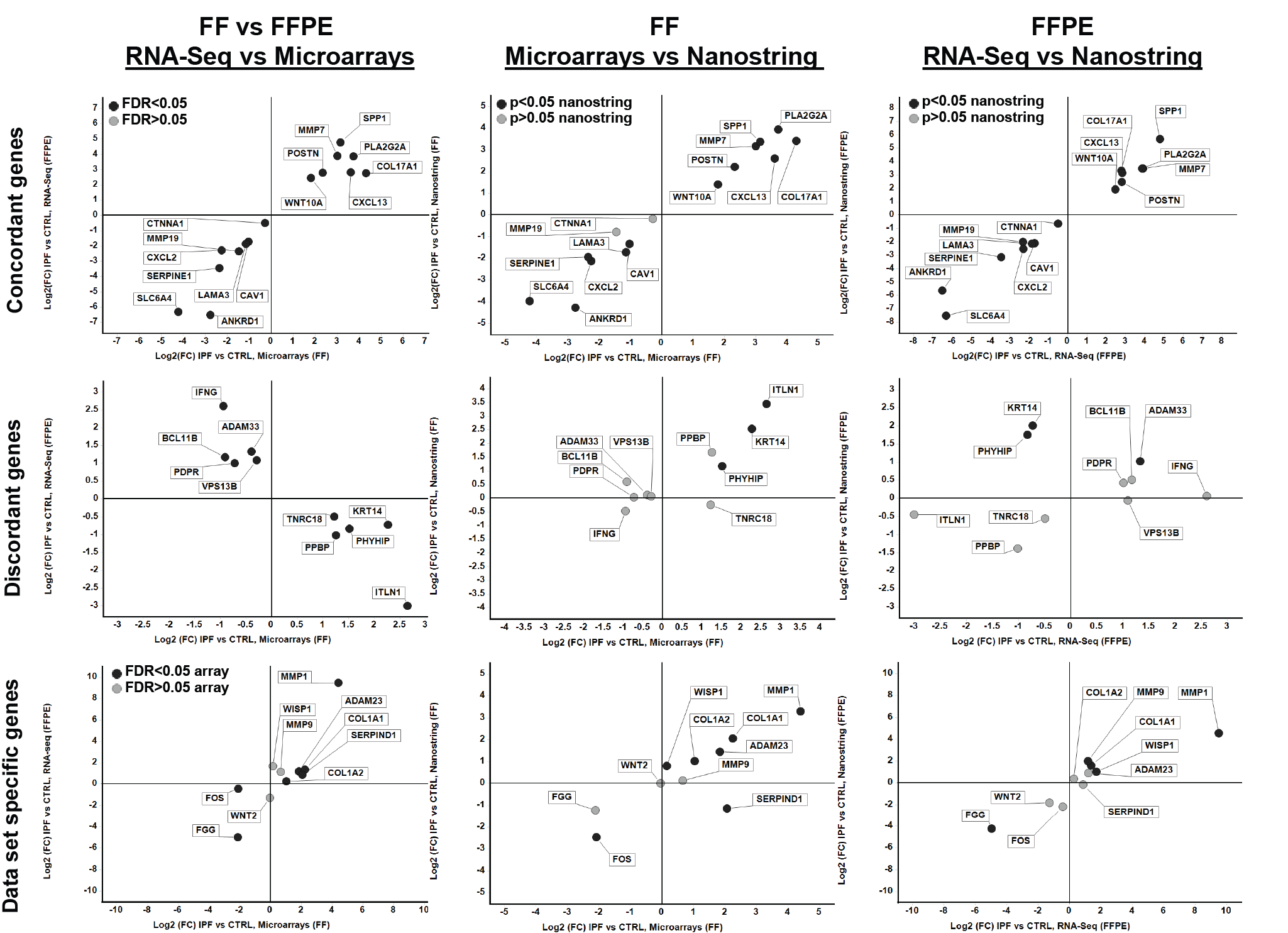

Supplement: Additional file 7: Figure S2. — Detailed results of NanoString nCounter validation. Upper 3 panels. Microarrays Log2(FC) IPF vs control (FF) was plotted on x axis and RNA-Seq Log2(FC) IPF vs control (FFPE) was plotted on y axis, than Nanostring Log2(FC) IPF vs control (FF) was plotted on y axis and Microarrays Log2(FC) IPF vs control (FF) was plotted on x axis, than Nanostring Log2(FC) IPF vs control (FFPE) was plotted on y axis and RNA-Seq Log2(FC) IPF vs control (FFPE) was plotted on x axis. 15 concordant genes between microarray and RNA-Seq were validated with NanoString. Gene names are labeled. FDR and p values are also labeled for each gene and technology. Similar to above, middle 3 panels present 10 discordant genes, and lower 3 panels present 10 genes in specific data set. (PNG 372 kb) [file 12890_2016_356_MOESM7_ESM.png]
